# Joint reconstruction of simultaneously acquired MR-PET data with multi sensor compressed sensing based on a joint sparsity constraint

**DOI:** 10.1186/2197-7364-1-S1-A26

**Published:** 2014-07-29

**Authors:** Florian Knoll, Thomas Koesters, Ricardo Otazo, Tobias Block, Li Feng, Kathleen Vunckx, David Faul, Johan Nuyts, Fernando Boada, Daniel K Sodickson

**Affiliations:** Bernard & Irene Schwartz Center for Biomedical Imaging, Department of Radiology, NYU School of Medicine, New York, New York USA; Department of Nuclear Medicine, KU Leuven, Leuven, Belgium; Siemens Medical Solutions USA, New York, USA

State-of-the-art MR-PET scanners allow simultaneous data acquisition. However, image reconstruction is performed separately and results are only combined at the visualization stage. PET images are reconstructed using a variant of EM and MR data are reconstructed using an inverse Fourier transform or iterative algorithms for parallel imaging or compressed sensing. We propose a new iterative joint reconstruction framework based on multi-sensor compressed sensing that exploits anatomical correlations between MR and PET.

Joint reconstruction is motivated by the fact that MR and PET are based on the same anatomy. High resolution MR information can be used to enhance the PET reconstruction and MR artifacts are not present in the PET image. Therefore a dedicated reconstruction can exploit the incoherence of artifacts in the joint space. Our approach uses a nonlinear constrained optimization problem. In each iteration OSEM enforces data consistency of the current solution with measured PET rawdata. An l1-norm based joint sparsity term exploits anatomical correlations. MR data consistency is enforced with the MR forward operator, consisting of coil sensitivity modulation and a (non-uniform) Fourier transform. Data were acquired on a clinical 3T MR-PET unit (Siemens Biograph mMR). 10 mCi 18F-FDG were injected followed by a 60min list mode scan. 3D MP-RAGE was used for MR data acquisition: TR=2300ms, TE=2.98ms, TI=900ms, FA=9°, acceleration factor 2, 24 ACS lines, 256 matrix, voxel size=1×1×1mm^3^, 192 slices.

Joint MR-PET reconstruction improves resolution in PET images when structures are aligned with MR. PET signal information cannot be improved in regions showing no distinctive MR contrast, but it is also not influenced falsely. The availability of simultaneously-acquired MR and PET data will also enable incorporation of dynamic correlations and motion correction into the joint reconstruction framework. We expect that this provides additional enhancements to the information content of multimodality studies.

